# MicroRNA signature of small‐cell lung cancer after treatment failure: impact on oncogenic targets by *miR‐30a‐3p* control

**DOI:** 10.1002/1878-0261.13339

**Published:** 2022-11-23

**Authors:** Kengo Tanigawa, Shunsuke Misono, Keiko Mizuno, Shunichi Asai, Takayuki Suetsugu, Akifumi Uchida, Minami Kawano, Hiromasa Inoue, Naohiko Seki

**Affiliations:** ^1^ Department of Pulmonary Medicine, Graduate School of Medical and Dental Sciences Kagoshima University Japan; ^2^ Department of Functional Genomics Chiba University Graduate School of Medicine Japan

**Keywords:** downstream neighbor of SON, microRNA signature, *miR‐30a‐3p*, small‐cell lung cancer, treatment failure

## Abstract

Small‐cell lung cancer (SCLC) is associated with a high mortality rate and limited treatment efficacy. We created a microRNA (miRNA) expression signature by RNA sequencing using specimens from patients with SCLC who had failed treatment. Forty‐nine miRNAs were downregulated in SCLC tissues and were candidate tumor‐suppressive miRNAs. In this signature, both guide and passenger strands were downregulated for five miRNAs (*miR‐30a*, *miR‐34b*, *miR‐34c*, *miR‐223*, and *miR‐4529*). Recent studies have revealed that passenger strands of miRNAs are involved in the molecular pathogenesis of human cancer. Although *miR‐30a‐5p* (the guide strand) has been shown to be a tumor‐suppressive miRNA in various types of cancers, *miR‐30a‐3p* (the passenger strand) function is not well characterized in SCLC cells. We investigated the functional significance of *miR‐30a‐3p* and oncogenic genes regulated by *miR‐30a‐3p* in SCLC cells. Ectopic expression assays showed that *miR‐30a‐3p* expression inhibited cell proliferation and induced cell cycle arrest and apoptosis in two SCLC cell lines. Furthermore, *in silico* database searches and gene expression assays identified 25 genes as putative targets of *miR‐30a‐3p* in SCLC cells. Luciferase reporter assays revealed that downstream neighbor of SON (*DONSON*) was directly regulated by *miR‐30a‐3p* in SCLC cells. Knockdown of *DONSON* induced cell cycle arrest in SCLC cells and DONSON overexpression were detected in SCLC clinical samples. Analyzing the regulatory networks of tumor‐suppressive miRNAs may lead to the identification of therapeutic targets in SCLC.

## Graphical abstract

microRNA (miRNA) expression signature for SCLC (small‐cell lung cancer) by RNA sequencing was created using clinical specimens after treatment failure. *miR‐30a‐3p* was downregulated and acted as a tumor‐suppressive miRNA in SCLC. Downstream neighbor of SON (*DONSON*) was directly regulated by *miR‐30a‐3p* and knockdown of *DONSON* induced suppression of SCLC progression.

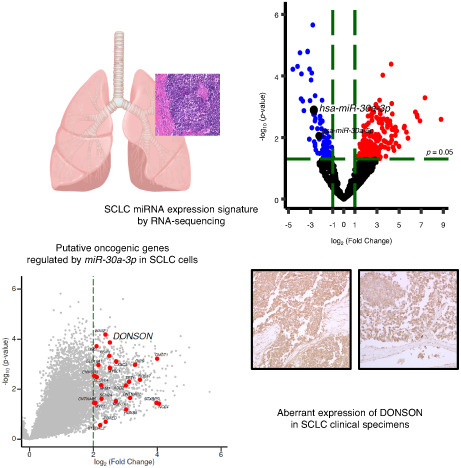

**Abbreviations**
DONSON: downstream neighbor of SON
ED‐SCLC: extensive disease‐SCLC
EZH2: enhancer of zeste homolog 2
GEO: Gene Expression Omnibus
miRNA: microRNA
NSCLC: non‐small‐cell lung cancer
OIP5: Opa interacting protein 5
qRT‐PCR: quantitative reverse transcription polymerase chain reaction
RCC: renal cell carcinoma
RISC: RNA‐induced silencing complex
SCLC: small‐cell lung cancer
TET1: ten‐eleven translocation methylcytosine dioxygenase 1

## 1 Introduction

In developed countries, lung cancer is the leading cause of cancer‐related deaths among men and women. Specifically, approximately, 2 100 000 people are diagnosed with lung cancer, and 1 800 000 patients die each year [1]. Lung cancer is generally divided into two types, non‐small‐cell lung cancer (NSCLC) and small‐cell lung cancer (SCLC), which accounts for 13–15% of all new lung cancer cases [2].

Due to the aggressive nature of SCLC, 80–85% of patients present with advanced or extensive disease‐SCLC (ED‐SCLC) at diagnosis [3]. The conventional first‐line treatment for ED‐SCLC is platinum‐based chemotherapy. Although patients with ED‐SCLC respond well to initial treatment, most patients develop recurrence or distant metastases within 1 year (median progression‐free survival: 5–6 months) [4]. Currently, no effective treatment has been found for patients with SCLC after initial treatment failure. Clarifying the molecular mechanisms through which cancer cells acquire drug resistance is an important issue for management of this disease.

In addition to protein‐coding genes, noncoding RNAs are also involved in the regulation of many biological processes, for example, cell proliferation, differentiation, apoptosis, and migration [5]. MicroRNAs (miRNAs) are noncoding RNAs that control gene transcription levels by binding to specific sites of target RNAs [6, 7]. Thus, they can fine‐tune physiological and pathological processes [8, 9]. Remarkably, a single miRNA can control a vast number of RNA transcripts. Therefore, aberrant expression of miRNAs can trigger the malignant transformation of human cells.

Many studies have revealed that aberrant miRNA expression occurs frequently in cancer cells and that miRNA expression is closely involved in cancer cell progression, metastasis, and drug resistance [10]. Furthermore, the latest genome analyses and miRNA databases have shown that many oncogenic molecular networks are controlled by miRNAs in cancer cells [11, 12]. Finding miRNAs that dysregulate gene expression in cancer cells is a logical starting point for cancer‐miRNA research.

Current RNA‐sequencing technology has made it possible to create genome‐wide miRNA signatures in a short amount of time [13, 14]. To date, a large number of miRNA expression signatures have been created using tissues and cells from various types of cancers [15, 16, 17]. Our group also created miRNA signatures using clinical specimens, for example, renal cell carcinoma (RCC), esophageal cancer, head and neck cancer, pancreatic cancer, and breast cancer [16, 17, 18, 19, 20]. RNA‐sequence‐based signatures have revealed that some passenger strands of miRNAs (e.g., *miR‐145‐3p*, *miR‐143‐5p*, *miR‐199a‐3p*, and *miR‐101‐5p*) act as tumor‐suppressive miRNAs by targeting oncogenic genes [16, 17, 18, 21]. The involvement of passenger strands of miRNAs in cancer pathogenesis is a new concept that is currently being explored in cancer research.

In this study, we created a new SCLC miRNA expression signature using autopsy samples from patients who experienced treatment failure. We analyzed the data to elucidate the molecular mechanisms of drug resistance in SCLC cells. In total, 49 miRNAs were downregulated in SCLC tissues, suggesting that these miRNAs were candidate tumor‐suppressive miRNAs in SCLC cells. Interestingly, among these miRNAs, both strands (guide and passenger strands) were downregulated for five miRNAs (i.e., *miR‐30a*, *miR‐34b*, *miR‐34c*, *miR‐223*, and *miR‐4529*).

Based on these signatures, we focused on *miR‐30a‐3p* (the passenger strand of the *miR‐30a*‐duplex). Our functional assays indicated that *miR‐30a‐3p* acted as a tumor‐suppressive miRNA in SCLC cells. Importantly, downstream neighbor of SON (*DONSON*) was found to be a direct target of *miR‐30a‐3p*, and aberrant expression of *DONSON* enhanced the aggressive SCLC cell phenotype. Here, we present a miRNA signature created from clinical specimens of SCLC after treatment failure. Analyzing the regulatory networks of antitumor miRNAs will lead to the identification of new therapeutic targets in SCLC.

## 2 Materials and methods

### 2.1 Clinical course of patients with SCLC and SCLC cell lines

**Fig. 1:**
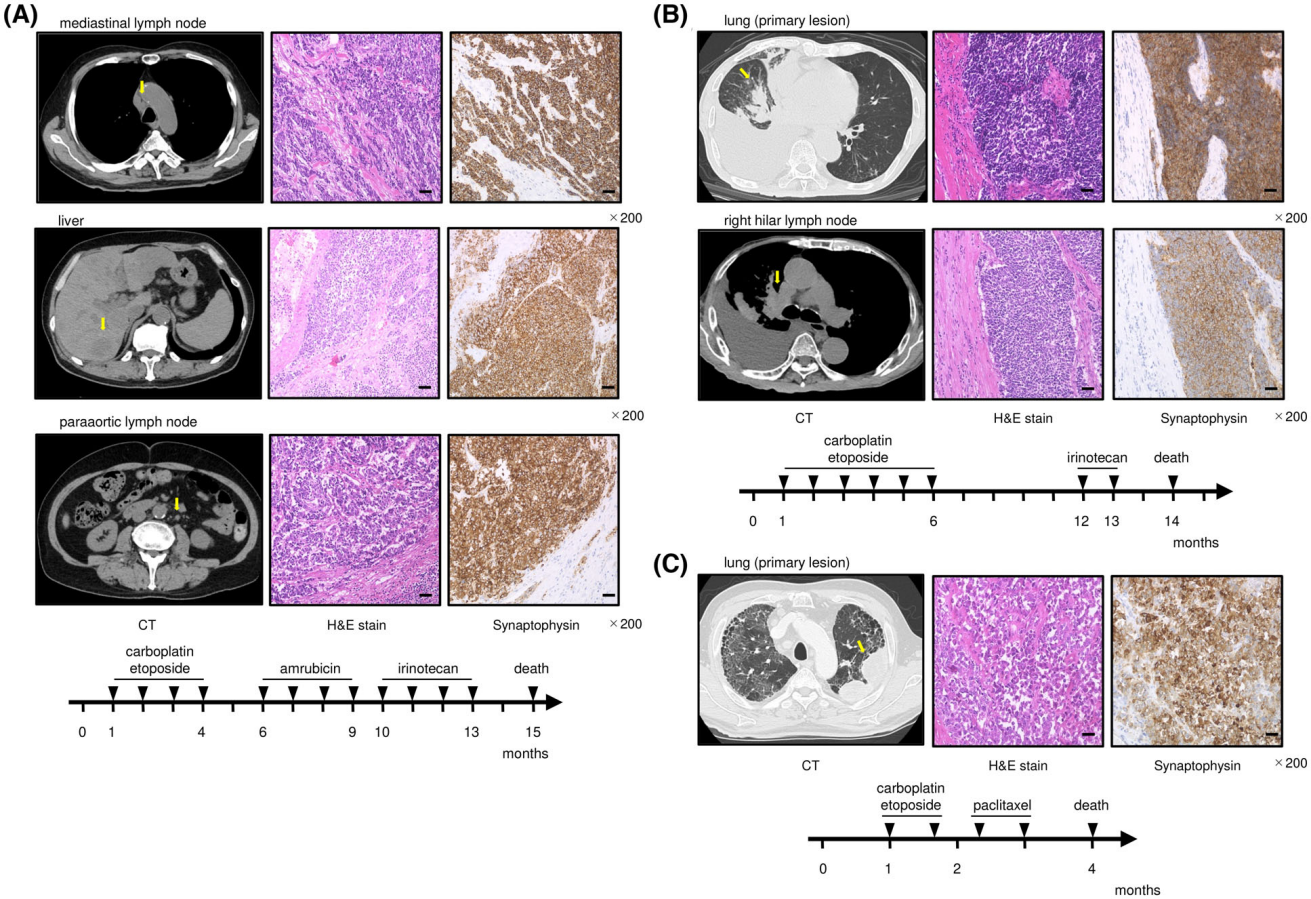
Clinical course in patients with SCLC whose tissues were used for generation of a miRNA expression signature. Computed tomography (CT), hematoxylin and eosin (HE) staining, and immunohistochemistry from autopsies and progression of disease in three patients with SCLC after diagnosis. (A) A 66‐year‐old man had edema of the face and breathing difficulties. He had a primary lung tumor at the hilum of the right lung and bone metastasis. He was diagnosed with SCLC by cytology and received chemotherapy (first line: Carboplatin and etoposide, second line: Amrubicin, third line: Irinotecan). After the third round of therapy, his condition deteriorated, and he died. Tissue specimens of the metastases (mediastinal lymph node, liver, Para‐aortic lymph node, and lesser curvature lymph node) were obtained from autopsy. (B) A 77‐year‐old man had a fever and was admitted to a hospital. A tumor located in the right lower lobe of the lung and multiple bone metastases were detected. A tissue sample from fiber bronchoscopy showed SCLC. He was treated with carboplatin and etoposide as first‐line therapy and irinotecan as second‐line therapy. He died of lung cancer 14 months after diagnosis. Tumor samples were obtained from the primary lesion (right lower lobe) and the right hilar lymph node metastasis. (C) A 65‐year‐old male patient saw a doctor for a persistent cough. The patient had a lung tumor in the left superior lobe and some metastases (left hilar lymph node, mediastinal lymph node, and multiple nodules in the right lung). A diagnosis by bronchoscopic biopsy revealed SCLC. First‐line chemotherapy with carboplatin and etoposide was performed, but progressive disease was observed after two courses. Although paclitaxel was initiated as second‐line chemotherapy, it showed no effect, and the patient passed away. Autopsy tissues were obtained from the left upper lobe (primary lesion) and the right intrapulmonary metastases. The arrows indicate a part of tissue samples used for miRNA expression signature. Scale bar: 50 μm in Fig. 1A–C.

Small‐cell lung cancer tissue specimens and normal lung tissue specimens were obtained from three patients, all of whom died from SCLC after treatment. The characteristics of the patients and the clinical causes of patients are presented in Fig. 1 and Table S1. This study's methodologies conformed to the standards set by the Declaration of Helsinki. We obtained written informed consent from all patients prior to participation in the study. The current study was approved by Ethics Committee on Epidemiological and its related Studies, Sakuragaoka Campus, Kagoshima University (Kagoshima, Japan; approval no. 210101Epi).

Two SCLC cell lines, SBC‐3 and H82, were used in this study. SBC‐3 cells were purchased from the Japanese Collection of Research Bioresources (Osaka, Japan). H82 cells were obtained from American Type Culture Collection (Manassas, VA, USA). Cell culture was carried out as described in our earlier reports [21, 22, 23].

### 2.2 Construction of the miRNA expression signature in advanced SCLC based on RNA‐sequencing

Total RNA was extracted from SCLC autopsy specimens. RNA sequencing was conducted using a HiSeq 2500 instrument (Illumina, Inc., San Diego, CA, USA) to evaluate miRNA expression in SCLC. The raw sequencing data were registered in Gene Expression Omnibus (GEO; GEO accession number: GSE176198).

### 2.3 Identification of oncogenic targets regulated by *miR‐30a‐3p* in SCLC cells

Expression profiles of genes from SBC‐3 cells transfected with *miR‐30a‐5p* and *miR‐30a‐3p* (GEO accession number: GSE139319) and microarray data from SCLC autopsy specimens (GEO accession number: GSE162102) were used in this screening. miRNA binding sites were predicted using TargetScanHuman ver.7.2 (http://www.targetscan.org/vert_72/).

### 2.4 RNA extraction and quantitative reverse transcription polymerase chain reaction (qRT‐PCR)

Total RNA from clinical specimens was isolated using TRI reagent (Cosmo Bio Co., Ltd., Tokyo, Japan). Total RNA was extracted from SCLC cells using Isogen II (NIPPON GENE Co., Ltd., Tokyo, Japan). Methods for evaluation of the quantity and quality of RNA and for qRT‐PCR have been described previously [23, 24]. TaqMan probes and primers are described in Table S2.

### 2.5 Transfection of miRNAs, small interfering RNAs (siRNAs), and plasmid vectors into SCLC cells

The miRNA and siRNA reagents in the current study are listed in Table S2. Opti‐MEM (Gibco, Carlsbad, CA, USA) and Lipofectamine RNAiMax Transfection Reagent (Invitrogen, Carlsbad, CA, USA) were used to transfect miRNAs and siRNAs into SCLC cells. Plasmid vectors were transfected into the cells using Lipofectamine 2000 Transfection Reagent (Invitrogen). The procedure for transfection was described in our previous reports [21, 22, 24].

### 2.6 Functional assays characterizing SCLC cells (cell proliferation, migration, apoptosis, and cell cycle assays)

The procedures for functional assays were described in our previous studies [21, 23, 24]. Briefly, XTT assays for assessment of growth were conducted with Cell Proliferation Kits (Biological Industries, Beit‐Haemek, Israel). Migration assays were performed using scratch wound healing assays. Apoptosis was evaluated using PE Active Caspase‐3 Apoptosis Kits (BD Biosciences, Franklin Lakes, NJ, USA). BD Cycletest Plus DNA Reagent Kits (BD Biosciences) were used to assess the cell cycle. Apoptosis assays and cell cycle analyses were performed with a BD FACSCelesta Flow Cytometer (BD Biosciences), and the results were analyzed using flowjo software (TreeStar, CA, USA).

### 2.7 Incorporation of *miR‐30a‐3p* target genes into the RNA‐induced silencing complex (RISC) by Ago2 immunoprecipitation

Incorporation of *miR‐30a‐3p* targets into the RISC was investigated using the Ago2 immunoprecipitation method. Twelve hours after *miR‐30a‐3p* transfection, the RISC was isolated using a human Ago2 miRNA isolation kit (FUJIFILM Wako Pure Chemical Corporation, Osaka, Japan), and the amount of incorporated *DONSON* was measured using qRT‐PCR. The procedure for Ago2 immunoprecipitation was described in our previous studies [23, 24].

### 2.8 Plasmid construction and dual‐luciferase reporter assays

The following two sequences were cloned into the psiCHECK‐2 vector (C8021; Promega, Madison, WI, USA): the wild‐type sequence of the 3′‐untranslated regions (UTRs) of *DONSON* and the deletion type, which lacked the *miR‐30a‐3p* target sites of *DONSON*. The procedures for transfection and dual luciferase reporter assays were provided in previous studies [23, 24].

### 2.9 Western blotting

The procedure for western blot analysis was described in previous studies [23, 24]. The primary antibodies are described in Table S2. The signal was developed using Amersham ECL Prime Western Blotting Detection Reagent (Cytiva, Marlborough, MA, USA). Chemiluminescence with FluorChem FC2 (Cell Biosciences, Santa Clara, CA, USA) was used to visualize western blotting bands. Western blotting was independently performed three times, and images were analyzed using imagej software.

### 2.10 Immunohistochemistry

A tissue microarray (catalog no.: LC811a; US Biomax, Inc. Derwood, MD, USA) was used for immunohistochemical staining. The primary antibodies used in this study are listed in Table S2. The procedure for immunostaining was described previously [23, 24].

### 2.11 Statistics

graphpad prism 7 (GraphPad Software, La Jolla, CA, USA) was used to conduct statistical analyses. One‐way analysis of variance and Tukey's *post hoc* test were used for multiple group comparisons.

## 3 Results

### 3.1 Small RNA sequencing of SCLC specimens and construction of miRNA signatures

In this study, RNA samples were obtained from autopsy specimens from patients with SCLC who showed treatment failure. Eight metastatic lesions were obtained from three patients, and we created a miRNA expression signature based on these samples (Table S1). The clinical characteristics of the three patients are summarized in Fig. 1A–C.

In RNA‐sequencing, we obtained between 441 473 462 and 615 202 138 reads. After filtering and trimming of the sequenced reads, between 9 428 865 and 20 351 100 miRNA reads larger than 19 nucleotides were mapped in the human genome (Table S3). Human genome‐matched sequence reads were divided into small RNAs according to their biological features (Table S3).

**Fig. 2:**
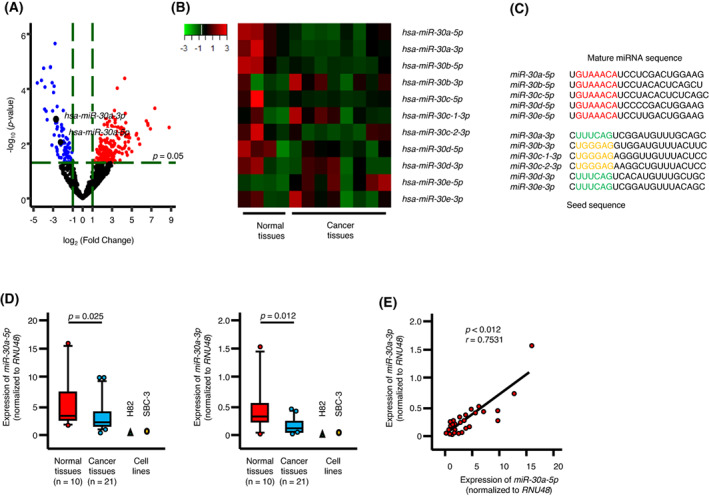
Expression levels of *miR‐30a‐5p* and *miR‐30a‐3p* in SCLC clinical specimens and cell lines. (A) Volcano plot of the miRNA expression signature determined by RNA sequencing. The log_2_ fold‐change (FC) is plotted on the *X*‐axis, and the −log_10_ *P*‐value is plotted on the *Y*‐axis. The blue points represent the downregulated miRNAs with an absolute −log_2_ FC ≥ 1 (FC = 2) and *P*‐value < 0.05. The red points represent the upregulated miRNAs with an absolute log_2_ FC ≥ 1 (FC = 2) and *P*‐value < 0.05. Cancer tissues: *n* = 8, Normal lung tissues: *n* = 4. glmLRT using edger (ver.3.8.6). (B) Heat map of the expression of *miR‐30* family members for Normal lung tissues and SCLC lung cancer tissues based on the SCLC miRNA signature. Cancer tissues: *n* = 8, Normal lung tissues: *n* = 4. (C) Mature sequences of *miR‐30* family members are indicated. Seed sequences of *miR‐30* family members are shown in red, green, and yellow. (D) the expression levels of *miR‐30a‐5p* and *miR‐30a‐3p* were evaluated in SCLC clinical tissues and cell lines (SBC‐3 and H82). The expression levels of these miRNAs were significantly reduced in cancer tissues (*P* < 0.001). The lower and upper hinges represent the 25th and 75th percentiles, respectively. The lower and upper whiskers represent the 10th and 90th percentiles, respectively. Cancer tissues: *n* = 21, Normal lung tissues: *n* = 10. Mann–Whitney *U* test. (E) Spearman's rank correlation showed positive correlations between the expression levels of *miR‐30a‐5p* and *miR‐30a‐3p* in clinical specimens (*r* = 0.7531, *P* = 0.012). Spearman's rank correlation.

We successfully identified significantly upregulated or downregulated miRNAs in SCLC tissues (Fig. 2A). In total, 49 downregulated miRNAs were identified (Table 1). Interestingly, among these dysregulated miRNAs, 18 were annotated as passenger strands of miRNAs in the miRBase database (Release 22, http://www.mirbase.org/).

**Table 1:** Downregulated miRNAs identified by RNA sequencing of SCLC clinical specimens. CPM, counts per million; FDR, false discovery rate; SCLC, small‐cell lung cancer.

MicroRNA	miRBase accession No.	Guide or passenger strand	Log_2_ fold change	Normalized read count (CPM)		*P* value	FDR
				Normal lung tissues	SCLC tissues		
*hsa‐miR‐34b‐3p*	MIMAT0004676	Guide strand	−4.61	155.35	4.47	6.06E‐05	0.020
*hsa‐miR‐605‐5p*	MIMAT0003273	Guide strand	−4.18	5.94	0.16	4.89E‐05	0.020
*hsa‐miR‐4529‐5p*	MIMAT0019236	Passenger strand	−3.96	6.47	0.31	1.76E‐05	0.011
*hsa‐miR‐4772‐3p*	MIMAT0019927	Guide strand	−3.86	2.92	0.06	5.53E‐04	0.088
*hsa‐miR‐504‐5p*	MIMAT0002875	Guide strand	−3.83	38.40	2.26	8.61E‐05	0.022
*hsa‐miR‐516a‐5p*	MIMAT0004770	Guide strand	−3.71	4.33	0.42	6.52E‐04	0.088
*hsa‐miR‐519a‐3p*	MIMAT0002869	Guide strand	−3.61	2.75	0.25	1.34E‐03	0.125
*hsa‐let‐7g‐3p*	MIMAT0004584	Passenger strand	−3.27	325.91	31.19	1.65E‐05	0.011
*hsa‐miR‐218‐1‐3p*	MIMAT0004565	Passenger strand	−3.27	35.99	3.38	1.58E‐05	0.011
*hsa‐miR‐1283*	MIMAT0005799	Guide strand	−3.12	0.91	0.04	1.14E‐02	0.268
*hsa‐miR‐223‐5p*	MIMAT0004570	Passenger strand	−3.10	13.33	1.98	5.98E‐05	0.020
*hsa‐miR‐4636*	MIMAT0019693	Guide strand	−3.05	5.68	0.55	6.36E‐04	0.088
*hsa‐miR‐29c‐5p*	MIMAT0004673	Passenger strand	−2.96	313.35	38.83	1.35E‐04	0.029
*hsa‐miR‐491‐5p*	MIMAT0002807	Guide strand	−2.90	38.23	4.30	8.01E‐05	0.022
*hsa‐miR‐4529‐3p*	MIMAT0019068	Guide strand	−2.89	2.36	0.23	4.83E‐03	0.180
*hsa‐miR‐181a‐3p*	MIMAT0000270	Passenger strand	−2.80	381.13	60.44	2.21E‐06	0.006
*hsa‐miR‐184*	MIMAT0000454	Guide strand	−2.75	10.51	1.54	1.76E‐03	0.130
*hsa‐miR‐30a‐3p*	MIMAT0000088	Passenger strand	−2.70	6020.30	983.06	1.29E‐03	0.125
*hsa‐miR‐4423‐5p*	MIMAT0019232	Passenger strand	−2.67	1.43	0.19	1.37E‐02	0.290
*hsa‐miR‐1247‐3p*	MIMAT0022721	Passenger strand	−2.62	12.81	2.76	1.69E‐03	0.130
*hsa‐miR‐3617‐5p*	MIMAT0017997	Guide strand	−2.61	5.23	0.76	2.89E‐03	0.138
*hsa‐miR‐4709‐5p*	MIMAT0019811	Passenger strand	−2.59	1.78	0.19	1.62E‐02	0.309
*hsa‐miR‐4703‐3p*	MIMAT0019802	Guide strand	−2.59	1.34	0.17	1.89E‐02	0.336
*hsa‐miR‐100‐3p*	MIMAT0004512	Passenger strand	−2.59	22.70	4.42	4.40E‐04	0.087
*hsa‐miR‐3199*	MIMAT0015084	Guide strand	−2.57	5.16	0.78	2.42E‐03	0.138
*hsa‐miR‐4536‐5p*	MIMAT0019078	Guide strand	−2.53	1.16	0.15	2.53E‐02	0.368
*hsa‐miR‐944*	MIMAT0004987	Guide strand	−2.38	13.17	2.09	5.17E‐03	0.183
*hsa‐miR‐34b‐5p*	MIMAT0000685	Passenger strand	−2.36	107.81	24.96	3.19E‐02	0.424
*hsa‐miR‐30a‐5p*	MIMAT0000087	Guide strand	−2.26	77110.51	18264.68	8.85E‐03	0.243
*hsa‐miR‐3667‐3p*	MIMAT0018090	Passenger strand	−2.25	0.67	0.07	6.66E‐02	0.624
*hsa‐miR‐521*	MIMAT0002854	Guide strand	−2.24	0.38	0.01	7.70E‐02	0.684
*hsa‐miR‐150‐5p*	MIMAT0000451	Guide strand	−2.22	367.12	78.55	1.12E‐02	0.268
*hsa‐miR‐6502‐5p*	MIMAT0025460	Guide strand	−2.18	1.66	0.34	3.28E‐02	0.424
*hsa‐miR‐4727‐5p*	MIMAT0019847	Passenger strand	−2.17	0.51	0.02	6.95E‐02	0.642
*hsa‐miR‐126‐3p*	MIMAT0000445	Guide strand	−2.15	5480.95	1462.45	2.05E‐03	0.135
*hsa‐miR‐223‐3p*	MIMAT0000280	Guide strand	−2.13	333.22	99.74	3.44E‐03	0.153
*hsa‐miR‐4804‐3p*	MIMAT0019985	Passenger strand	−2.12	0.64	0.06	7.80E‐02	0.689
*hsa‐miR‐29b‐2‐5p*	MIMAT0004515	Passenger strand	−2.11	10.31	2.46	2.60E‐03	0.138
*hsa‐miR‐548v*	MIMAT0015020	Guide strand	−2.08	0.39	0.01	8.79E‐02	0.727
*hsa‐miR‐1258*	MIMAT0005909	Guide strand	−2.08	0.88	0.15	7.93E‐02	0.692
*hsa‐miR‐203a‐3p*	MIMAT0000264	Guide strand	−2.05	268.47	83.10	2.86E‐03	0.138
*hsa‐miR‐516b‐5p*	MIMAT0002859	Guide strand	−2.05	0.29	0.00	8.80E‐02	0.727
*hsa‐miR‐574‐3p*	MIMAT0003239	Guide strand	−2.05	2537.12	595.00	9.77E‐03	0.252
*hsa‐miR‐34c‐3p*	MIMAT0004677	Passenger strand	−2.04	13.64	2.81	5.34E‐02	0.546
*hsa‐miR‐6507‐5p*	MIMAT0025470	Guide strand	−2.03	0.64	0.09	9.81E‐02	0.764
*hsa‐miR‐145‐3p*	MIMAT0004601	Passenger strand	−2.01	183.32	50.60	5.19E‐03	0.183
*hsa‐miR‐4800‐3p*	MIMAT0019979	Guide strand	−2.01	0.42	0.03	1.10E‐01	0.804
*hsa‐miR‐548h‐5p*	MIMAT0005928	Guide strand	−2.01	0.47	0.08	1.24E‐01	0.856
*hsa‐miR‐34c‐5p*	MIMAT0000686	Guide strand	−2.00	394.48	109.00	6.51E‐02	0.616

Our signature revealed that some members of the *miR‐30* family were downregulated in SCLC tissues (Fig. 2B). Both strands of miRNAs derived from pre‐*miR‐30a* (*miR‐30a‐5p* and *miR‐30a‐3p*) were significantly downregulated in SCLC tissues (Fig. 2A). Normalized read counts of *miR‐30a* indicated the highest elevation levels in normal lung tissue (Table 1). In this study, we focused on *miR‐30a‐5p* (the guide strand) and *miR‐30a‐3p* (the passenger strand) and investigated the functional significance of these miRNAs in SCLC cells. Mature sequences of *miR‐30* family members are shown in Fig. 2C.

### 3.2 Expression levels of *miR‐30‐5p* and *miR‐30a‐3p* in SCLC specimens and cell lines

To confirm our miRNA signature, we evaluated the expression levels of *miR‐30a‐5p* and *miR‐30a‐3p* in SCLC tissues and normal lung tissues. Both *miR‐30a‐5p* (*P* = 0.025) and *miR‐30a‐3p* (*P* = 0.012), were significantly downregulated in SCLC tissues (Fig. 2D). Moreover, the expression levels of these two miRNAs were positively correlated (Fig. 2E). Additionally, we confirmed that the expression levels of these miRNAs were very low in both SBC‐3 and H82 cells (Fig. 2D).

### 3.3 Restoration of *miR‐30a‐5p* and *miR‐30a‐3p*: Effects on cell proliferation, migration, cell cycle arrest, and apoptosis cells in SCLC cells

**Fig. 3:**
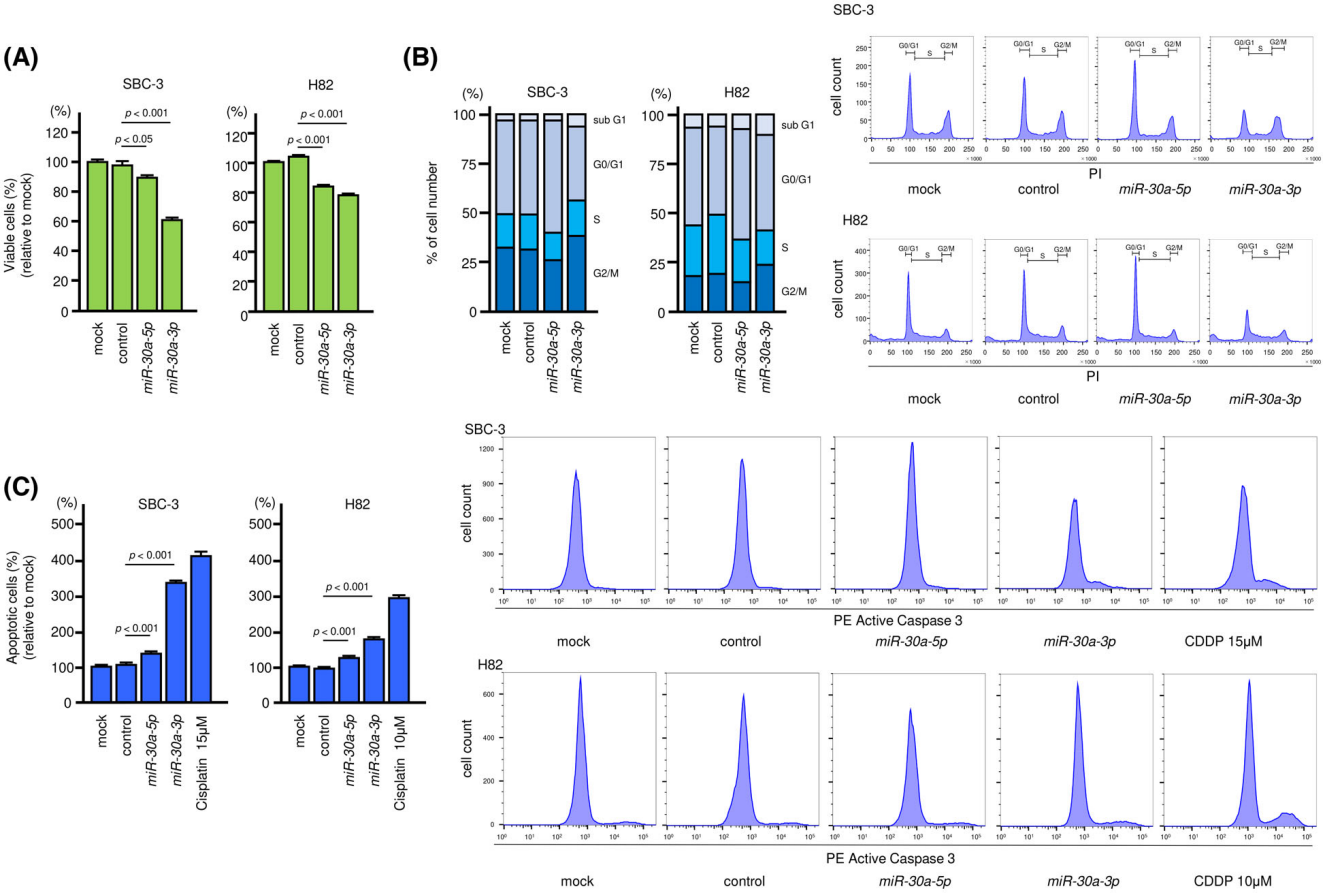
Tumor‐suppressive roles of *miR‐30a‐5p* and *miR‐30a‐3p* in SCLC cells. Functional assays of *miR‐30a‐5p* and *miR‐30a‐3p* in SBC‐3 and H82 cells. (A) Cell proliferation was assessed using XTT assay 72 h after transfection with mature miRNAs. Data are mean ± SD. *n* = 3. One‐way ANOVA and Tukey tests for *post hoc* analysis. (B) Flow cytometry analysis of cell cycle status after transfection with *miR‐30a‐5p* or *miR‐30a‐3p*. Data of stacked bar graphs are mean. Representative images were shown. *n* = 3. (C) The proportion of apoptotic cells at 72 h after transfection with *miR‐30a‐5p* or *miR‐30a‐3p* was evaluated by flow cytometry. CDDP was used as a positive control. Data are mean ± SD. Representative images were shown. *n* = 3. One‐way ANOVA and Tukey tests for *post hoc* analysis.

To identify the tumor‐suppressive functions of *miR‐30a‐5p* and *miR‐30a‐3p* in SCLC cells, we performed ectopic expression assays in SBC‐3 and H82 cells. Proliferation assays showed that growth was reduced in SCLC cells after transfection with *miR‐30a‐5p* or *miR‐30a‐3p* compared with that in cells transfected with control miRNA (Fig. 3A). Analysis of cell migration ability was performed using wound healing assays. Ectopic expression of *miR‐30a‐3p* significantly suppressed the migration ability of SBC‐3 cells (Fig. S1). Cell cycle assays demonstrated that increased proportions of cells resided in the G_0_/G_1_ phase after induction of *miR‐30a‐5p* expression and in the G_2_/M phase after induction of *miR‐30a‐3p* expression in the two cell lines (Fig. 3B). We further investigated the induction of apoptosis after *miR‐30a‐5p* or *miR‐30a‐3p* expression. In apoptosis assays, ectopic expression of both miRNAs increased the percentage of apoptotic cells in both SCLC cell lines (Fig. 3C).

### 3.4 Identification of putative target genes regulated by *miR‐30a‐3p* in SCLC cells

Some genes controlled by *miR‐30a‐5p* are closely associated with the molecular pathogenesis of human cancers. By contrast, *miR‐30a‐3p* has not been carefully examined in SCLC cells. Our recent studies showed that passenger strands of miRNAs are closely involved in the molecular pathogenesis of human cancers. Therefore, in this study, we focused on *miR‐30a‐3p* (the passenger strand derived from pre‐*miR‐30a*).

**Fig. 4:**
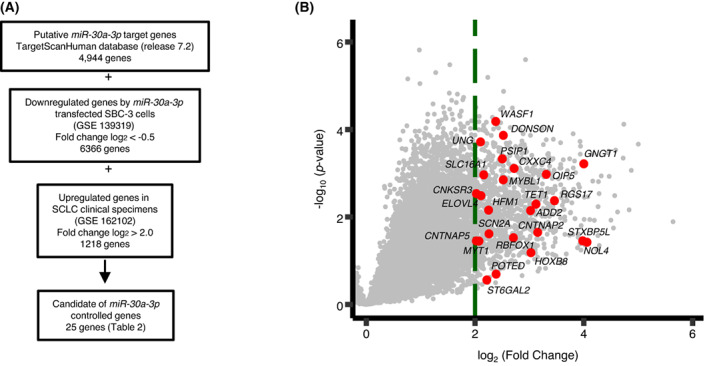
Flowchart for identifying oncogenic targets subject to *miR‐30a‐3p* regulation in SCLC cells. (A) To identify genes controlled by *miR‐30a‐3p* in SCLC cells, we used the TargetScanHuman (release 7.2) database and two of our original gene expression profiles, *miR‐30a‐3p* transfected SBC‐3 cells (GSE139319) and the molecular signature of patients with SCLC after treatment failure (GSE162102). In total, 25 genes were identified as possibly controlled by *miR‐30a‐3p* in SCLC cells. (B) Volcano plot showing upregulated genes in SCLC clinical specimens. The 25 candidate genes are shown in red circles. Cancer tissues: *n* = 8, Normal lung tissues: *n* = 4.

Based on the TargetScanHuman database (http://www.targetscan.org/vert_72/), 4944 candidate genes had *miR‐30a‐3p* binding sites in their 3′‐UTRs. Additionally, we used genome‐wide gene expression analysis to detect downregulated genes in *miR‐30a‐3p* transfected SBC‐3 cells (GEO accession number: GSE139319), and we created a gene expression signature using SCLC clinical specimens (GEO accession number: GSE162102). We then merged the data to narrow down the candidate targets of *miR‐30a‐3p* regulation in SCLC cells. Thus, 25 oncogenic targets were identified (Table 2). Our analysis strategy is shown in Fig. 4A. Expression levels of *miR‐30a‐3p* targets (25 genes) are plotted in Fig. 4B.

**Table 2:** Candidate target genes regulated by *miR‐30a‐3p*.

Entrez GeneID	Gene symbol	GeneName	Total sites	8mer sites	7mer‐m8 sites	7mer‐A1 sites	SBC‐3 *miR‐30a‐3p* transfectant Log_2_ fold change	SCLC tissues microarray Log_2_ fold change
8715	*NOL4*	Nucleolar protein 4	2	0	1	1	−0.60	4.06
2792	*GNGT1*	Guanine nucleotide binding protein (G protein), gamma transducing activity polypeptide 1	3	1	1	1	−1.05	4.00
9515	*STXBP5L*	Syntaxin binding protein 5‐like	1	0	1	0	−0.81	3.98
26 575	*RGS17*	Regulator of G‐protein signaling 17	1	1	0	0	−0.73	3.46
11 339	*OIP5*	Opa interacting protein 5	2	0	0	2	−0.79	3.31
26 047	*CNTNAP2*	Contactin associated protein‐like 2	1	0	0	1	−1.50	3.15
80 312	*TET1*	TET methylcytosine dioxygenase 1	1	0	1	0	−0.66	3.12
3218	*HOXB8*	Homeobox B8	1	0	0	1	−0.98	3.03
119	*ADD2*	Adducin 2 (beta)	3	0	1	2	−1.45	3.02
80 319	*CXXC4*	CXXC finger protein 4	2	0	1	1	−0.60	2.72
54 715	*RBFOX1*	RNA binding protein, fox‐1 homolog (*C. elegans*) 1	1	0	0	1	−0.62	2.71
29 980	*DONSON*	Downstream neighbor of SON	1	0	1	0	−0.71	2.52
4603	*MYBL1*	v‐myb avian myeloblastosis viral oncogene homolog‐like 1	1	1	0	0	−0.84	2.52
11 168	*PSIP1*	PC4 and SFRS1 interacting protein 1	1	1	0	0	−0.74	2.50
317 754	*POTED*	POTE ankyrin domain family, member D	2	0	2	0	−1.41	2.39
8936	*WASF1*	WAS protein family, member 1	2	0	1	1	−0.52	2.38
6326	*SCN2A*	Sodium channel, voltage‐gated, type II, alpha subunit	1	0	0	1	−0.63	2.25
164 045	*HFM1*	HFM1, ATP‐dependent DNA helicase homolog (*S. cerevisiae*)	1	0	0	1	−1.78	2.25
84 620	*ST6GAL2*	ST6 beta‐galactosamide alpha‐2,6‐sialyltranferase 2	1	0	1	0	−0.74	2.22
6566	*SLC16A1*	Solute carrier family 16 (monocarboxylate transporter), member 1	4	0	0	4	−0.54	2.16
6785	*ELOVL4*	ELOVL fatty acid elongase 4	2	0	1	1	−0.75	2.11
7374	*UNG*	Uracil‐DNA glycosylase	2	1	1	0	−0.57	2.10
4661	*MYT1*	Myelin transcription factor 1	1	0	0	1	−0.76	2.07
154 043	*CNKSR3*	CNKSR family member 3	7	2	2	3	−0.54	2.02
129 684	*CNTNAP5*	Contactin‐associated protein family member 5	1	0	1	0	−1.14	2.02

### 3.5 Direct regulation of *DONSON* by *miR‐30a‐3p* in SCLC cells

**Fig. 5:**
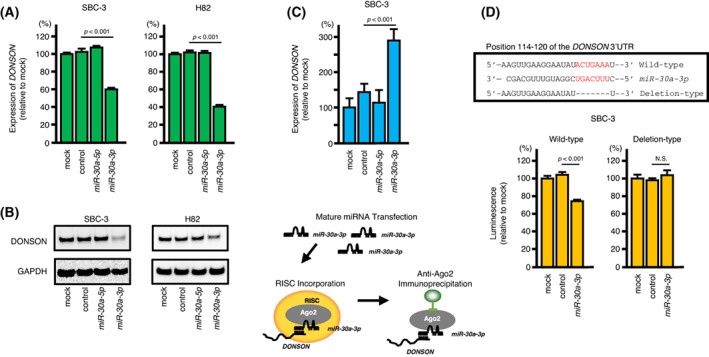
Direct regulation of *DONSON* expression by *miR‐30a‐3p* in SCLC cells. (A) qRT‐PCR showing significantly reduced expression of *DONSON* mRNA 72 h after *miR‐30a‐3p* transfection in SBC‐3 and H82 cells. *GAPDH* was used as an internal control. Data are mean ± SD. *n* = 3. One‐way ANOVA and Tukey tests for *post hoc* analysis. (B) Western blot analysis showing reduced expression of DONSON protein 72 h after *miR‐30a‐3p* transfection in SCLC cells. GAPDH was used as an internal control. Representative images were shown. *n* = 3. (C) Incorporation of *DONSON* into the RISC in SBC‐3 cells. The incorporated *DONSON* was prepared to high purity using immunoprecipitation with anti‐human Ago2 monoclonal antibodies. *DONSON* incorporation was quantified using qRT‐PCR. The lower panel shows a schematic diagram depicting *DONSON* incorporation into the RISC. Data are mean ± SD. *n* = 3. One‐way ANOVA and Tukey tests for *post hoc* analysis. (D) a putative *miR‐30a‐3p* binding site predicted within the 3′‐UTR of *DONSON* by TargetScanHuman database analysis (upper panel). Dual luciferase reporter assays showed reduced luminescence activity after cotransfection of the wild‐type *DONSON* 3′‐UTR sequence (containing the *miR‐30a‐3p* binding site) with *miR‐30a‐3p* in SCLC cells (lower panel). Normalized data were calculated as the *Renilla*/firefly luciferase activity ratio (N.S., not significant). Data are mean ± SD. *n* = 3. One‐way ANOVA and Tukey tests for *post hoc* analysis.

Among the putative target genes regulated by *miR‐30a‐3p*, we focused on *DONSON* because our previous study showed that its aberrant expression enhanced cancer cell aggressive phenotypes in RCC cells [20]. However, the function of *DONSON* in SCLC remains unknown. The expression levels of DONSON mRNA and protein were significantly reduced by *miR‐30a‐3p* transfection in SCLC cells (Fig. 5A,B). Full images of western blots are presented in Fig. S2. Next, we evaluated whether *DONSON* was actually incorporated into the RISC in SCLC cells. Our data demonstrated that DONSON was incorporated into the RISC in *miR‐30a‐3p* transfected SCLC cells (Fig. 5C). Finally, we examined whether *miR‐30a‐3p* bound directly to the 3′‐UTR of *DONSON* using dual‐luciferase reporter assays. According to the TargetScanHuman database, one putative binding site was annotated in the 3′‐UTR of *DONSON* (Fig. 5D). Our data showed that luminescence intensities were significantly reduced by cotransfection of *miR‐30a‐3p* and vectors carrying the *miR‐30a‐3p* binding site in the 3′‐UTR of *DONSON* (Fig. 5D). By contrast, cotransfection of *miR‐30a‐3p* and vectors without *miR‐30a‐3p* binding sites (deleted *miR‐30a‐3p* binding site) did not reduce luminescence intensities (Fig. 5D). These data indicated that *miR‐30a‐3p* bound directly to *DONSON* and controlled the expression of *DONSON* in SCLC cells.

### 3.6 Effects of *DONSON* knockdown on cell proliferation, migration, cell cycle arrest, and apoptosis

**Fig. 6:**
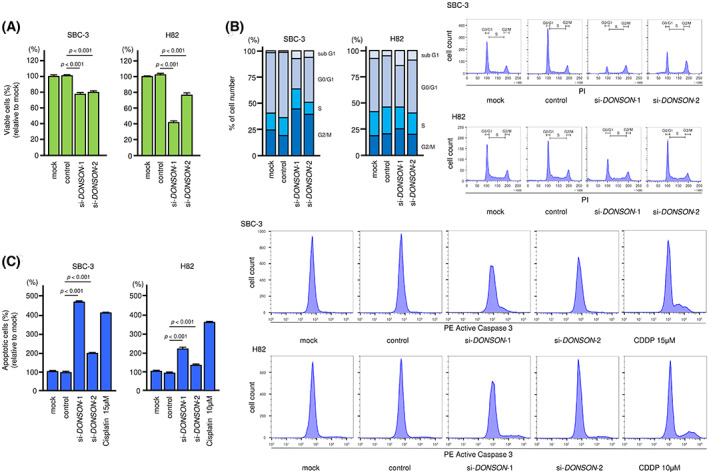
Effects of *DONSON* knockdown in SCLC cells. Functional assays assessing the effects of si‐*DONSON*‐1 and si‐*DONSON*‐2 transfection into SBC‐3 and H82 cells. (A) Cell proliferation was assessed using XTT assays 72 h after transfection of si‐*DONSON*‐1 and si‐*DONSON*‐2 into SCLC cells. Data are mean ± SD. *n* = 3. One‐way ANOVA and Tukey tests for *post hoc* analysis. (B) Cell cycle status after transfection of si‐*DONSON*‐1 and si‐*DONSON*‐2 into SCLC cells, as characterized by flow cytometry. Data of stacked bar graphs are mean. Representative images were shown. *n* = 3. (C) Proportions of apoptotic cells after transfection (72 h) of si‐*DONSON*‐1 and si‐*DONSON*‐2, as measured by flow cytometry. CDDP was used as a positive control. Data are mean ± SD. Representative images were shown. *n* = 3. One‐way ANOVA and Tukey tests for *post hoc* analysis.

To investigate the oncogenic roles of *DONSON* in SCLC cells, we conducted *DONSON* knockdown assays using siRNAs in SCLC cells. First, we evaluated the knockdown efficiencies of si‐*DONSON* in SCLC cells. Transient transfection with two types of siRNAs significantly reduced DONSON mRNA and protein expression in SCLC cells (Fig. S3A,B). Full images of western blots are presented in Fig. S3B. Cell proliferation assays showed that si‐*DONSON* transfection reduced the growth of SCLC cells compared with that in cells transfected with control siRNA (Fig. 6A). Analysis of cell migration ability was performed using wound healing assays. Transient transfection with siRNAs targeting *DONSON* significantly suppressed the migration ability of SBC‐3 cells (Fig. S4). Additionally, cell cycle assays demonstrated that the proportions of cells in the sub‐G_1_ phase and G_2_/M phase were increased after *DONSON* knockdown in SBC‐3 cells. In H82 cells, sub‐G_1_ phase cells were increased, and si‐*DONSON*‐1 increased the proportion of cells in G_2_/M phase (Fig. 6B). In apoptosis assays, *DONSON* knockdown increased the percentage of apoptotic cells in both SCLC cell lines (Fig. 6C).

### 3.7 Expression of DONSON in SCLC clinical specimens

**Fig. 7:**
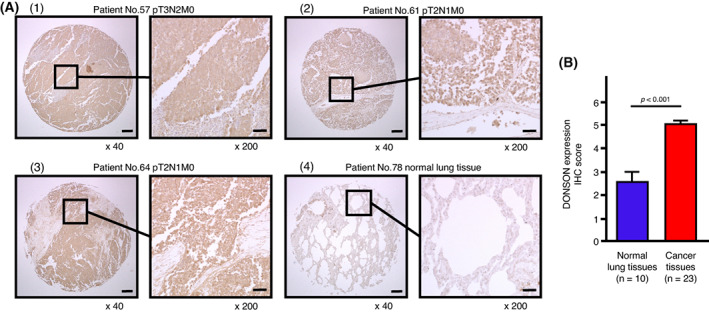
Expression of DONSON in clinical SCLC tissues. (A1–3) Immunohistochemical staining of DONSON in SCLC tissues. Overexpression of DONSON was observed in the cytoplasm of cancer cells. (A4) Immunohistochemical staining of DONSON in normal lung tissues. Low expression of DONSON was observed in normal cells. Scale bar: 200 μm (low magnification), 50 μm (high magnification) in Fig. 7A. Representative images were shown. Cancer tissues: *n* = 23, Normal lung tissues: *n* = 10. (B) Comparison of the scoring of DONSON expression in clinical lung specimens. DONSON expression was significantly higher in SCLC tissues than in normal lung tissues. Data are mean ± SEM. Mann–Whitney *U* test.

Finally, we investigated the expression levels of DONSON in SCLC clinical specimens by immunohistochemistry. DONSON was overexpressed in several cancer lesions. In particular, the cytoplasm of cancer cells was heavily stained (Fig. 7A1–3). By contrast, DONSON staining was very weak in normal lung tissue (Fig. 7A4). The protein expression of DONSON was scored (Fig. 7B), and we found that DONSON was overexpressed in SCLC tissues compared with normal lung tissues. The characteristics of the patients from whom samples were collected for immunostaining are shown in Table S4. Moreover, DONSON expression was investigated using FFPE specimens (six specimens) from SCLC patients who failed treatment (Table S5). Overexpression of DONSON was detected in primary and metastatic cancer lesions (Fig. S5).

## 4 Discussion

In SCLC cells, disruption of the cell cycle is often observed, and metastasis to distant sites is a common feature. In addition, SCLC cells easily acquire resistance to platinum‐based chemotherapy during the course of treatment. Few treatments are approved for recurrence and the distant metastases of the disease [25, 26, 27]. The molecular mechanisms through which SCLC cells acquire drug resistance remain unclear. Notably, several reports have evaluated miRNAs involved in drug resistance using drug‐resistant SCLC cell lines [28, 29, 30]. A previous study showed that ectopic expression of *miR‐134*, *miR‐379*, and *miR‐495* enhanced resistance to multiple drugs, including cisplatin, etoposide, and doxorubicin [28]. Another study showed that *miR‐7* was significantly downregulated in drug‐resistant SCLC cells compared with parental cells [29]. Notably, multidrug resistance‐associated protein 1 (*MRP1*) was shown to be directly controlled by *miR‐7* [29]. Aberrantly expressed miRNAs in drug‐resistant cell lines can provide important hints for exploring the molecular networks involved in drug resistance.

It is difficult to obtain clinical SCLC specimens. Thus, genomic analysis in SCLC has not been conducted satisfactorily. Previously, we created an SCLC miRNA signature using a PCR‐based array system. We successfully identified 35 downregulated miRNAs in a primary SCLC lesion and metastatic lesions (liver and brain). Those lesions were compared with noncancerous tissues [15]. Recently, we had the opportunity to obtain clinical specimens from patients with ED‐SCLC who experienced treatment failure. In this study, an SCLC miRNA signature was created by RNA sequencing, and we successfully identified 49 tumor‐suppressive miRNA candidates.

Very few large‐scale miRNA signatures using SCLC clinical specimens have been created. Recently, using the GEO database, differential expression of genes and miRNAs in SCLC was reported [31]. Compared with our newly created signature, the following downregulated miRNAs were common: *miR‐30a‐5p*, *miR‐30a‐3p*, *miR‐126‐3p*, *miR‐145‐3p*, *miR‐150‐5p*, and *miR‐223‐3p*. Among these miRNAs, our previous studies showed that *miR‐145‐3p* and *miR‐150‐5p* function as tumor‐suppressive miRNAs in lung squamous cell carcinoma and lung adenocarcinoma through their targeting of several oncogenes [21, 22, 23]. Notably, *miR‐145‐3p* is the passenger strand derived from the *miR‐145*‐duplex. In the original concept of miRNA biogenesis, the passenger strand was thought to be broken down inside the cell and to have no function [32]. Recently, our studies and other reports have revealed that both miRNAs derived from a single miRNA‐duplex can act as tumor‐suppressive miRNAs. Moreover, the two miRNA strands cooperatively regulate several oncogenic targets and pathways [21, 24, 33, 34]. The SCLC signature reported herein contains many miRNA passenger strands. The function of passenger strands of miRNAs in SCLC remains unclear. Analysis of miRNA passenger strands and their target genes may provide insights into novel pathogenic pathways in SCLC cells.

We have continued to analyze the functional roles of passenger strands of miRNAs and their control of molecular networks in cancer cells. In this study, we selected *miR‐30a‐3p* because few reports have described this miRNA in SCLC cells. Our functional assays showed that *miR‐30a‐3p* expression attenuated the proliferation of SCLC cells and demonstrated that this miRNA was a tumor‐suppressive miRNA. Previous studies have also demonstrated that *miR‐30a‐3p* exerts tumor‐suppressive functions in several types of cancers, including gastric cancer, lung adenocarcinoma, and RCC [35, 36, 37]. We recently showed that *miR‐30a‐3p* is a tumor‐suppressive miRNA that regulates various oncogenes in pancreatic ductal adenocarcinoma cells [19]. Interestingly, several genes regulated by *miR‐30a‐3p* affect the prognosis of patients with pancreatic cancer [19]. More recently, *miR‐30a‐3p* was shown to suppress the expression of matrix metalloproteinase‐2 and ‐9 and to reduce the invasive ability of bladder cancer cells [38]. Interestingly, *miR‐30a‐3p* enhanced the chemosensitivity of bladder cancer cells to cisplatin through suppression of protective autophagy [38].

Many studies have shown that abnormal CpG methylation and histone modifications can dysregulate miRNA expression in cancer cells [39]. Moreover, aberrant expression of several epigenetic regulators (e.g., polycomb repressive complexes, DNA methyl‐transferases, and histone deacetylases) can also affect miRNA expression in cancer cells [39]. Enhancer of zeste homolog 2 (*EZH2*) is a pivotal epigenetic regulator, and its overexpression was detected in a wide range of cancers, including SCLC [40]. *EZH2* has also been shown to control the expression of *miR‐30d* through promoter‐binding activity [41]. Thus, further studies are needed to assess the involvement of epigenetic modifications of *miR‐30a* expression.

In this study, we identified 25 genes that were regulated by *miR‐30a‐3p* in SCLC cells. Identifying genes regulated by *miR‐30a‐3p* should facilitate the identification of new molecular pathways underlying the pathogenesis of this disease. Opa interacting protein 5 (*OIP5*) was initially cloned by yeast two‐hybrid analysis as a protein interacting with Opa proteins [42]. Expression of OIP5 enhances cell cycle progression through interaction with retinoblastoma protein [43]. Aberrant expression of OIP5 has been reported in several types of cancer cells and is associated with poor patient prognosis in colorectal cancer, gastric cancer, esophageal cancer, and lung cancer [44, 45]. In hepatocellular carcinoma, OIP5 activates AKT oncogenic signaling and enhances cancer cell metastasis [46]. Numerous genetic and epigenetic alterations are required to transform normal cells into cancer cells [47]. Improper DNA methylation can shut down tumor‐suppressive genes, leading to tumor development [48]. For example, Ten‐eleven translocation methylcytosine dioxygenase 1 (*TET1*) is a member of the TET family and is closely involved in DNA demethylation [49, 50]. Previous studies have shown that TET1 is bound to promoter regions of tumor‐suppressive genes and enhances their expression [51, 52]. In lung cancer, oncogenic epidermal growth factor receptor‐mediated signals inhibit the expression of tumor‐suppressive genes through TET1 inhibition [53]. Furthermore, wild‐type p53 blocks *TET1* expression in lung cancer cells, whereas mutant p53 induces *TET1* expression, and overexpression of TET1 acts as an oncogene [54].

In this study, we focused on *DONSON* and found that this target was directly regulated by tumor‐suppressive *miR‐30a‐3p* in SCLC cells. *DONSON* is a replisome component involved in fork stabilization during genome replication [55]. Moreover, our recent study showed that *DONSON* is targeted by tumor‐suppressive *miR‐101‐5p* in RCC [20]. Aberrant expression of *DONSON* enhances the malignant features of cancer cells, and its overexpression is a strong independent predictor of unfavorable overall survival in patients with RCC [20, 56]. In SCLC cells, we observed that siRNA‐mediated knockdown of *DONSON* induced cell cycle arrest. This is the first report showing that the *miR‐30a‐3p*/*DONSON* axis is closely involved in the aggressive features of SCLC cells.

## 5 Conclusions

In conclusion, we reported a new RNA‐sequence‐based SCLC miRNA signature using clinical specimens from patients who experienced treatment failure. We demonstrated the presence of 49 downregulated miRNAs in SCLC tissues. Analysis of our signature revealed that several passenger strands of miRNAs were significantly downregulated in SCLC tissues. This is the first report demonstrating that ectopic expression of *miR‐30a‐3p* (the passenger strand) attenuated SCLC cell aggressiveness. These results suggested that *miR‐30a‐3p* acts as tumor suppressor. In total, 25 genes were identified as *miR‐30a‐3p* targets in SCLC cells. In particular, we demonstrated that *DONSON*, which we identified from analyses of genes regulated by *miR‐30a‐3p*, may be a novel therapeutic target in SCLC cells.

## Conflict of interest

The authors declare no conflict of interest.

## Author contributions

KM, HI, and NS conceived the study and designed the experiments. KT, SM, AU, and NS wrote the manuscript. KT, SM, MK, and AU performed the experiments. KT, SM, SA, TS, and AU analyzed the data. All authors read and approved the manuscript.

### Peer review

The peer review history for this article is available at https://publons.com/publon/10.1002/1878‐0261.13339.

## Supporting information


**Fig. S1.** Inhibition of migration by *miR‐30a‐3p* in SBC‐3 cells. Figure shows the images of cell migration assay by *miR‐30a‐3p*. Scale bar: 200μm. Data are mean ± SD. Representative images were shown. n = 3. One‐way ANOVA and Tukey tests for post‐hoc analysis.

**Fig. S2.** Full‐size images of the western blots shown in Figure 5. Figure shows the full‐size images of western blot by *miR‐30a‐5p* and *miR‐30a‐3p*. Data are mean ± SD. n = 3. One‐way ANOVA and Tukey tests for post‐hoc analysis.

**Fig. S3.** Efficiencies of *DONSON* knockdown by siRNAs in SCLC cells. A: RT‐PCR was performed to validate the mRNA expression of *DONSON*. Data are mean ± SD. n = 3. One‐way ANOVA and Tukey tests for post‐hoc analysis. B: The full‐size images of western blot using siRNAs were shown. Data are mean ± SD. n = 3. One‐way ANOVA and Tukey tests for post‐hoc analysis.

**Fig. S4.** Inhibition of migration by si‐*DONSON* in SBC‐3 cells. Figure shows the images of cell migration assay. Scale bar: 200μm. Data are mean ± SD. Representative images were shown. n = 3. One‐way ANOVA and Tukey tests for post‐hoc analysis.

**Fig. S5.** Expression of DONSON in refractory SCLC clinical specimens. Immunohistochemical staining of DONSON was conducted using FFPE specimens from SCLC patients who failed treatment. Scale bar: 50μm. n = 6.

**Table S1.** A. Clinical features of patients with SCLC from whom autopsy tissues were collected. B. Sample collection sites for miRNA sequencing.

**Table S2.** Reagents used in this study.

**Table S3.** Annotations of reads aligned to small RNAs.

**Table S4.** Characteristics of the patients from whom samples were collected for immunostaining.

**Table S5.** Characteristics of cases in Figure S5.

## Data Availability

The data that support the findings of this study are openly available online in GSE176198 (https://www.ncbi.nlm.nih.gov/geo/query/acc.cgi?acc=GSE176198), GSE139319 (https://www.ncbi.nlm.nih.gov/geo/query/acc.cgi?acc=GSE139319), and GSE162102 (https://www.ncbi.nlm.nih.gov/geo/query/acc.cgi?acc=GSE162102) datasets.
