# Supplementary figures and images for: MicroRNA signature of small‐cell lung cancer after treatment failure: impact on oncogenic targets by miR‐30a‐3p control
Source: Mol Oncol. 2022 Nov 23;17(2):328–43. doi: 10.1002/1878-0261.13339 (PMC9892828; doi:10.1002/1878-0261.13339)

## SBC-3

0h

24h

mock

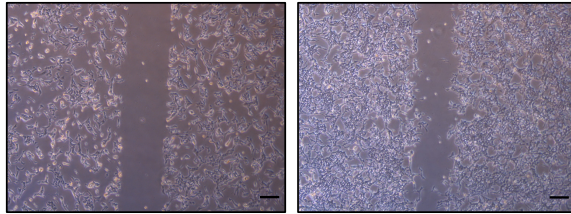

control

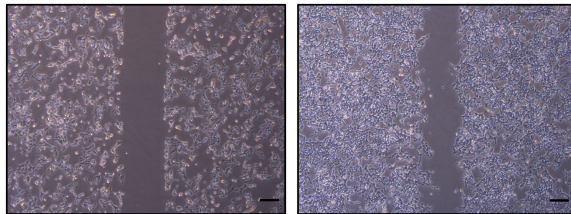

*miR-30a-3p*

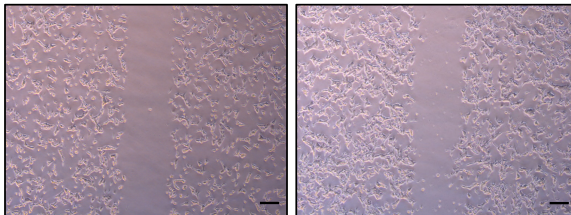

( $\times 40$ )

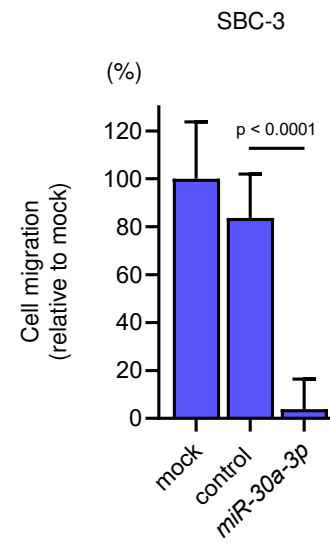

Supplement: Supplementary file 1 — Fig. S1. Inhibition of migration by miR‐30a‐3p in SBC‐3 cells. Figure shows the images of cell migration assay by miR‐30a‐3p. Scale bar: 200μm. Data are mean ± SD. Representative images were shown. n = 3. One‐way ANOVA and Tukey tests for post‐hoc analysis. [file MOL2-17-328-s002.pdf]

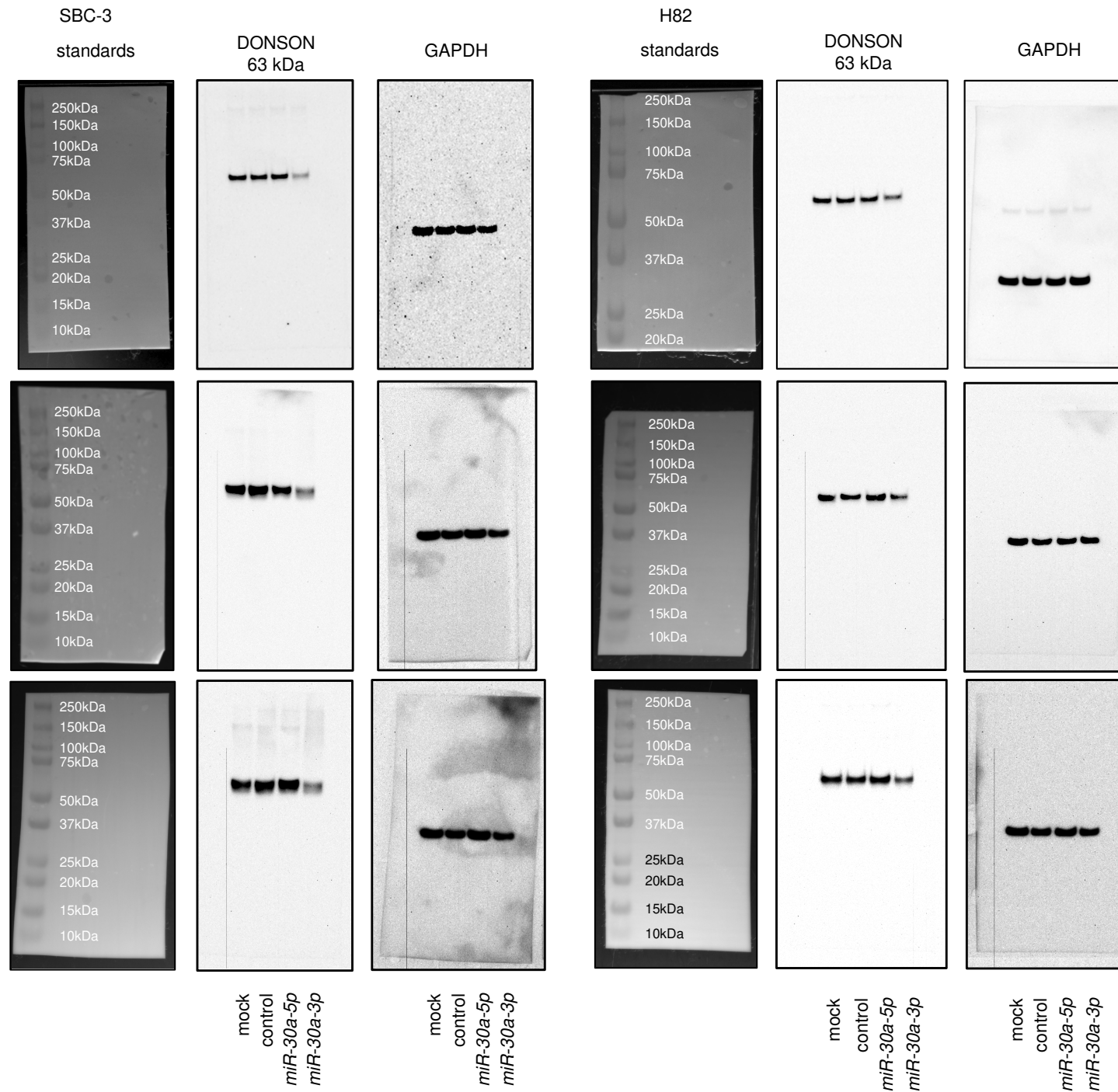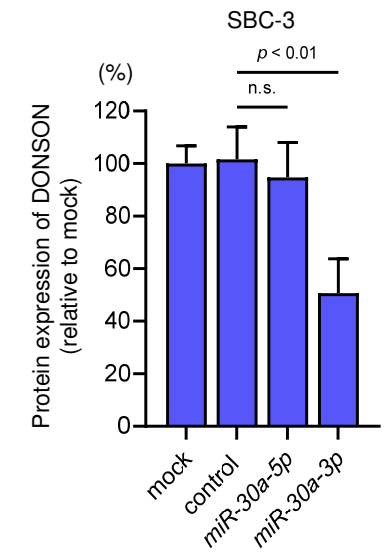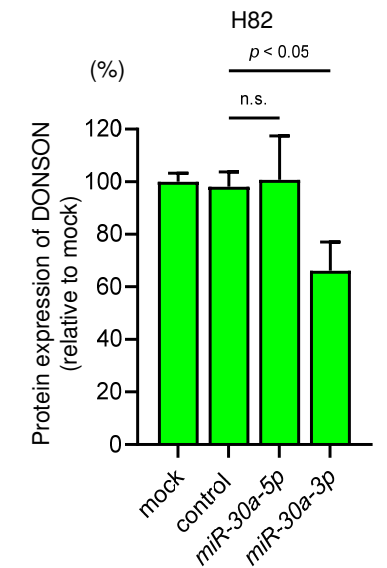

Supplement: Supplementary file 2 — Fig. S2. Full‐size images of the western blots shown in Figure 5. Figure shows the full‐size images of western blot by miR‐30a‐5p and miR‐30a‐3p. Data are mean ± SD. n = 3. One‐way ANOVA and Tukey tests for post‐hoc analysis. [file MOL2-17-328-s001.pdf]

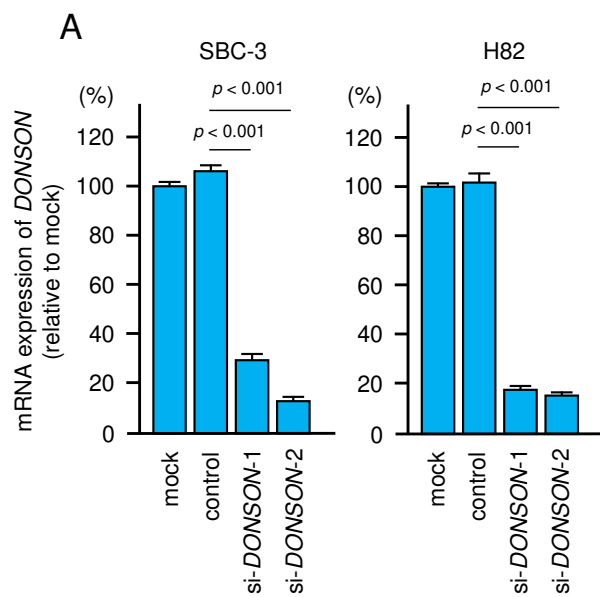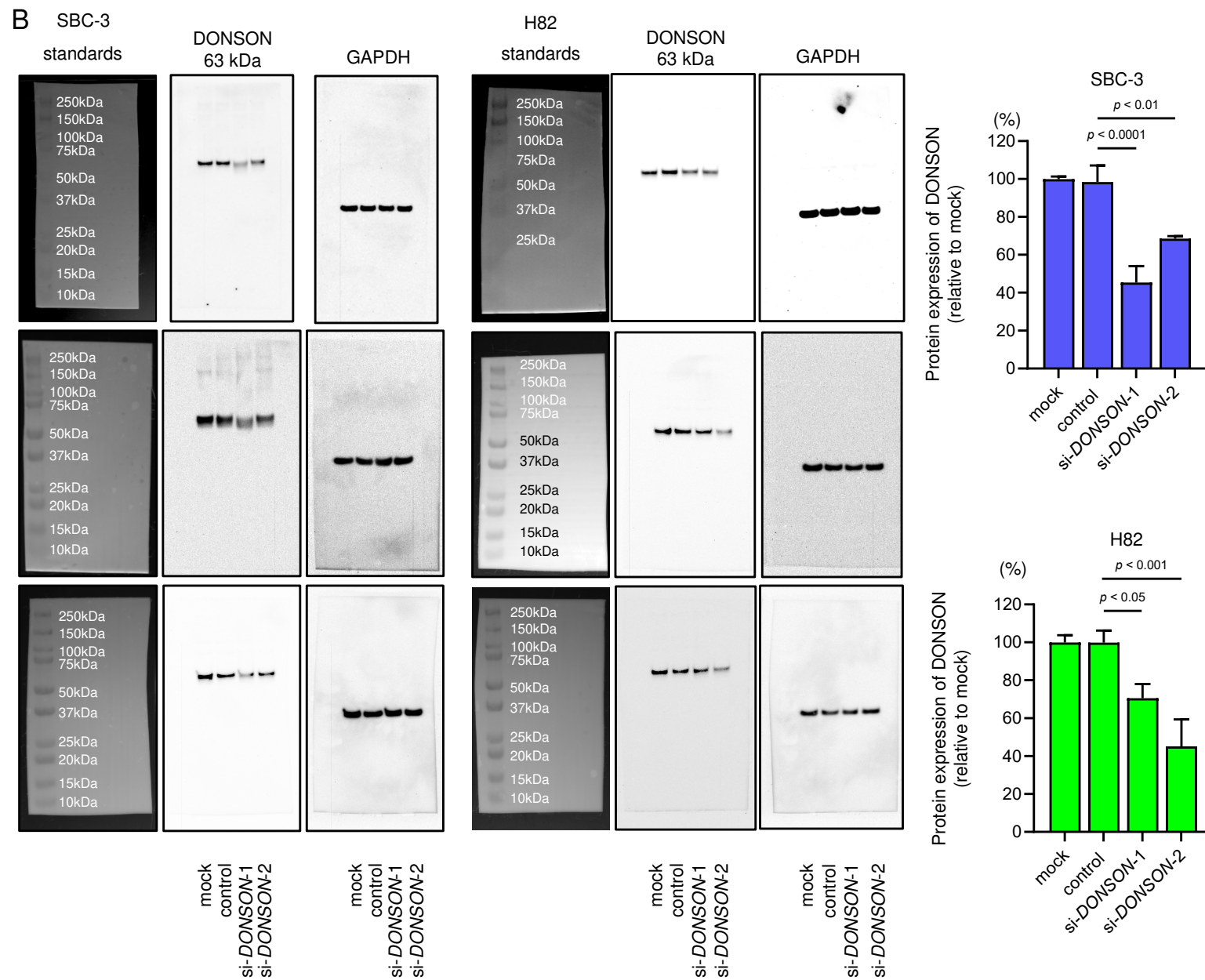

Supplement: Supplementary file 3 — Fig. S3. Efficiencies of DONSON knockdown by siRNAs in SCLC cells. A: RT‐PCR was performed to validate the mRNA expression of DONSON. Data are mean ± SD. n = 3. One‐way ANOVA and Tukey tests for post‐hoc analysis. B: The full‐size images of western blot using siRNAs were shown. Data are mean ± SD. n = 3. One‐way ANOVA and Tukey tests for post‐hoc analysis. [file MOL2-17-328-s006.pdf]

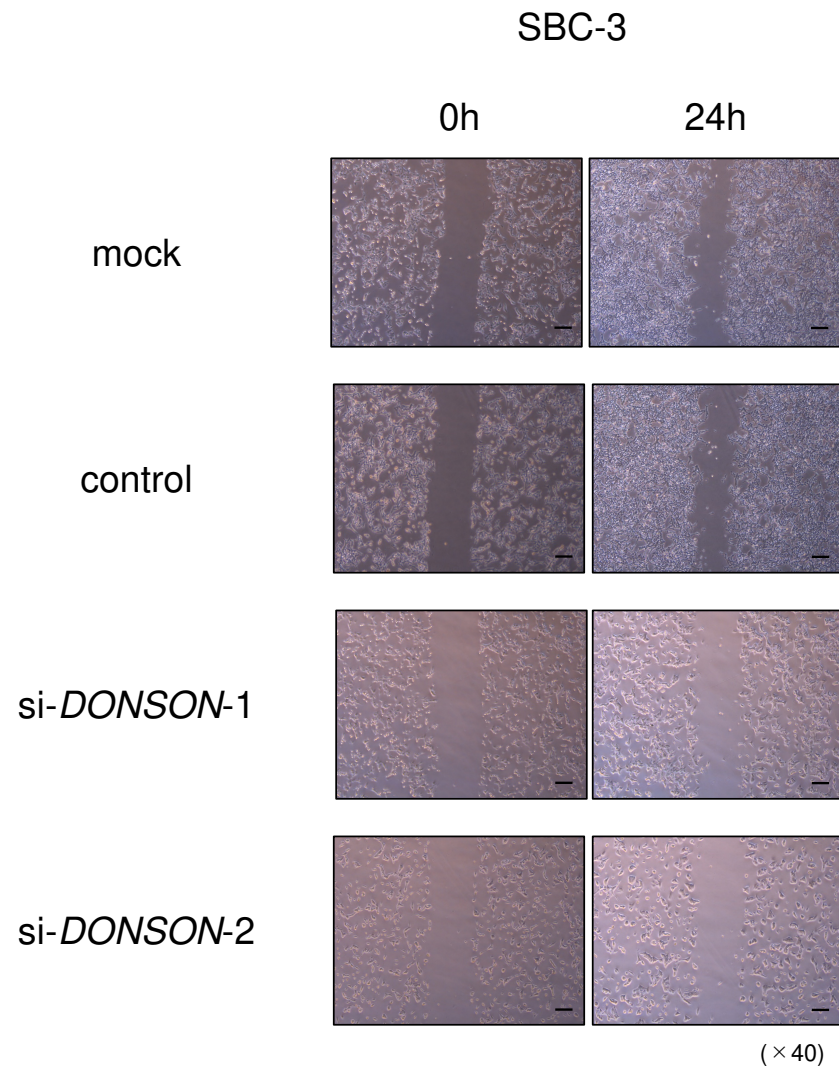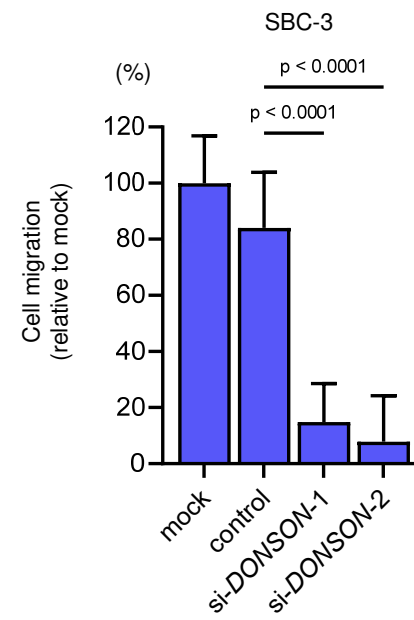

Supplement: Supplementary file 4 — Fig. S4. Inhibition of migration by si‐DONSON in SBC‐3 cells. Figure shows the images of cell migration assay. Scale bar: 200μm. Data are mean ± SD. Representative images were shown. n = 3. One‐way ANOVA and Tukey tests for post‐hoc analysis. [file MOL2-17-328-s004.pdf]
