# Supplementary material for: MicroRNA signature of small‐cell lung cancer after treatment failure: impact on oncogenic targets by miR‐30a‐3p control
Source: Mol Oncol. 2022 Nov 23;17(2):328–43. doi: 10.1002/1878-0261.13339 (PMC9892828; doi:10.1002/1878-0261.13339)
Supplement: Supplementary file 5 — Fig. S5. Expression of DONSON in refractory SCLC clinical specimens. Immunohistochemical staining of DONSON was conducted using FFPE specimens from SCLC patients who failed treatment. Scale bar: 50μm. n = 6. [file MOL2-17-328-s007.pdf]

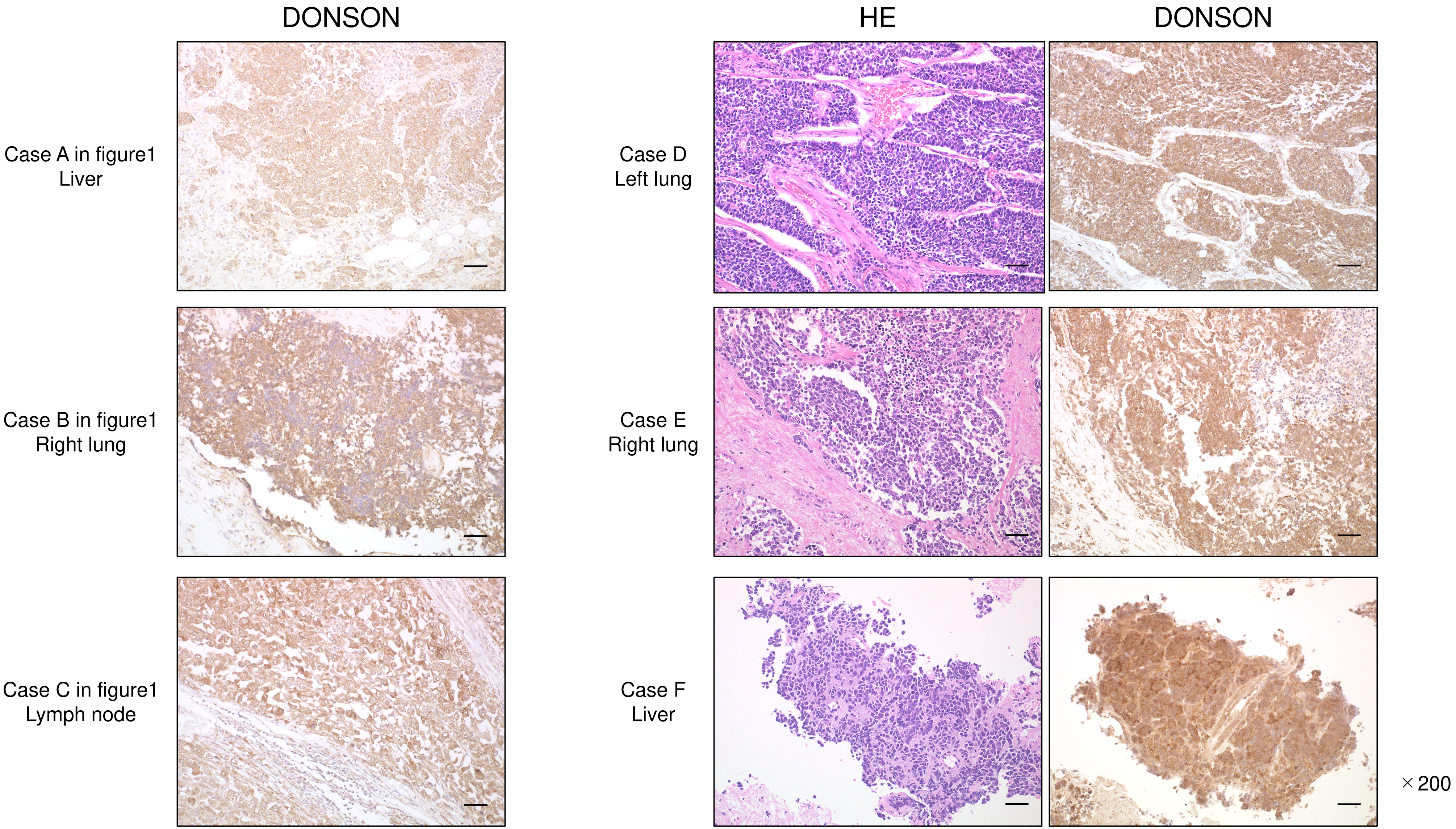
DONSON
HE
DONSON
Case A in figure1
Liver
Case B in figure1
Right lung
Case C in figure1
Lymph node
Case D
Left lung
Case E
Right lung
Case F
Liver
×200
